# The Influence of Laparscopic Sleeve Gastrectomy on Male Erectile Function among Morbid Obese Patients: an Observational Study

**DOI:** 10.1007/s11695-025-08376-5

**Published:** 2025-12-04

**Authors:** Mahmoud Azhary, Mohamed H. Fahmy, Ahmed Mohammed Salah Eldeen Othman Elansary, Ehab Fathy Ahmed, Mohamed Ahmed AbdElSalam, Ahmed Eid Aziz, Mohamed Elshal, Ahmed Maher Abd Elmonim

**Affiliations:** Cairo University, Cairo, Egypt

**Keywords:** LSG, Erectile dysfunction, Obesity, Quality of life, Sex hormones

## Abstract

Obesity is caused by caloric intake exceeding over time, Obesity has multiple effects on the capacity to get and sustained an erection. Obesity and ED in males appear to be linked in many studies. Obesity is a significant risk factor for the progress of ED, which is a serious Clinical issue. This study was performed to assess the impact of weight loss induced by laparoscopic sleeve gastrectomy (LSG) on male sex hormones and erectile function among morbid obese male patients. The study was done over 32 morbidly obese married male cases who underwent LSG for weight reduction after doing assessments during their preoperative preparation. All subjects were evaluated by 4 questionnaires and serum hormone profiles pre-and postoperatively. Our study showed statistically significant improvement in the erectile function, and the Erection Hardness Score among morbid obese men undergoing LSG after 6 months of operation. Also showed statistically significant increasing levels of testosterone and decreasing levels of Estradiol (E2) after 6 months of operation. LSG is effective with significant enhancement of the Bariatric Quality of Life and Bariatric Body Image Satisfaction.

**Graphical Abstract:**

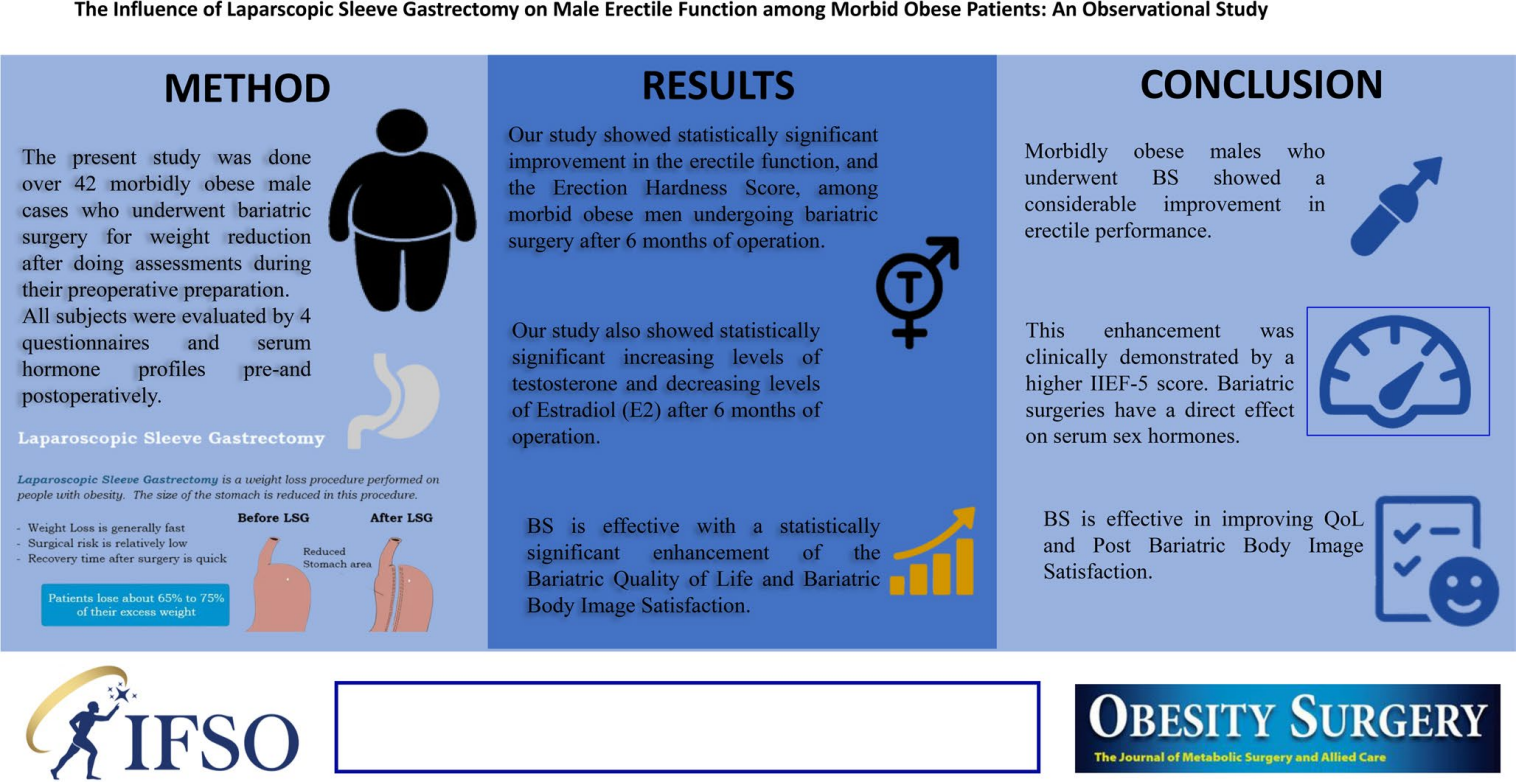

**Supplementary Information:**

The online version contains supplementary material available at 10.1007/s11695-025-08376-5.

## Introduction

Obesity is a non-communicable disease that is increasing in incidence worldwide. It is marked by excessive or abnormal fat accumulation that has an adverse impact on health, reduces life expectancy, and increases the risk of medical conditions (such as type 2 diabetes mellitus (T2DM), dyslipidemia, hypertension, ischemic heart disease, stroke, and certain cancer types) and/or overall mortality [1].

The main pillars of initial management of obesity are to minimize caloric intake and increase energy expenditure through; diet control, regular physical exercise, and pharmacotherapy to decrease fat absorption from the gut and reduce appetite or elevate caloric consumption.

However, obese individuals are more likely to have erectile dysfunction (ED), with 79% of ED cases also being overweight [2].

Obesity can trigger organic erectile dysfunction due to related metabolic syndrome, which includes an association of more than three of the factors that follow: high serum triglyceride levels, hypertension, DM, abdominal obesity, and low levels of high-density lipoprotein [3]. Obesity, on the other hand, may contribute to psychological ED because obese people experience melancholy, anxiety, low self-esteem, and poor body image. As a result, individuals believe themselves sexually unappealing and unappealing, leading them to avoid potential or real sexual interactions [4]. Excess body fat has also been linked to a variety of endocrine disorders. The most prevalent process is enhanced peripheral aromatization of testosterone into estrogen by the aromatase enzyme, followed by suppression of the hypothalamic-hypophyseal-gonadal [5].

Recent research has demonstrated that BS for morbidly obese cases not only substantially decreases weight in a short duration, but also greatly enhances sexual function. Nevertheless, the research number and and the majority of them are cohort investigations, the impact of weight loss surgery on several dimensions of male sexual performance remains controversial [6].

This study is designed to assess the influence of weight loss induced by BSs on male sex hormones and erectile function among morbid obese male patients.

## Materials and methods

### Study Participants

This prospective observational study included 32 patients presenting to a bariatric outpatient clinic. All patients underwent LSG for weight reduction after doing assessments in The Andrology Department during their preoperative preparation, and they were followed up after 6 months during the period from January 2022 to December 2023, fulfilling inclusion and exclusion criteria.

Cases with the following criteria were included:

All morbidly married obese male patients (BMI ≥ 35 kg/m2). Age ranging from 18 to 60 years old. I am willing to give consent and commit to a long-term follow-up schedule. Patients are generally fit for surgical procedures and anesthesia.

Cases with the following characteristics were excluded:

Extremes of age (< 18 and 60 years old).

Any contraindication to surgery, e.g., ASA class IV. History of abdominopelvic surgeries. History of receiving radiotherapy or chemotherapy for cancer treatment.

Chronic heavy smoker (smoking index ≥ 400).

Alcoholic patients with neurological diseases, either central or peripheral.

Patients with comorbidities or medications causing erectile dysfunction as sever hypertension and diabetes mellitus.

Cases with Peyronie’s disease or penile fibrosis. Patients with major psychotic disorders.

### Sample Size

The sample size was assessed employing “Clincalc sample size calculator”; considering the primary outcome, the mean (SD) of the preoperative IIEF score is 17.5 (6.05), and the mean five-item IIEF score of individuals who underwent BS increased by 5.66 points with alpha error 0.05 and the power of the study is 85% [7]. So, the sample size included 42 patients.

### Perioperative Care

Patients were made aware and consented to the purpose of the study, and they all understood and agreed to the process. All Cases received a normal preoperative evaluation, which included a detailed personal, medical, and surgical history.

Subjects received dietary advice and followed a reduced-calorie diet for 1–3 weeks before surgery based on their preoperative BMI. All co-morbidities that increased perioperative risk were managed to the greatest extent possible before surgery. Laboratory studies and imaging were performed, including a complete blood picture, HbA1c, and fasting blood sugar. Clinical chemistry (Creatinine, urea, ALT, AST, and serum albumin). Prothrombin concentration and time. Abdominal ultrasonography, chest X-ray, electrocardiogram, and echocardiography. Serum hormone profile [Testosterone (Total), Testosterone (Free), Estradiol E2].

### Data Collection

All selected patients were subjected to full medical history (Age, BMI, Drugs, Operations, Diseases) and clinical examination (both general and local examination). In addition, all subjects were evaluated by 4 questionnaires [International Index of Erectile Function (IIEF-5) Questionnaire, Erection Hardness Score (EHS) Questionnaire, Bariatric Quality of Life Index Questionnaire, Post Bariatric Body Image Satisfaction Questionnaire] and serum hormone profile [Testosterone (Total), Testosterone (Free), Estradiol E2] at two different occasions [i.e. preoperatively (Baseline) and postoperatively (After 6 months)]. All these assessments were reevaluated for all patients after 6 months after the operation to detect the impact of BS on erectile function.

### IIEF-5 Questionnaire

All cases completed the Arabic version of the IIEF questionnaire in a structured interview, which included five categories (IIEF- Desire, IIEF-Erectile Function, IIEF-Orgasm Function, IIEF-Intercourse Satisfaction, and IIEF-Total Satisfaction). Each domain received a score from 1 to 5. The IIIEF-5 score is the total of questions 1 through 5. The lowest score is 5, while the greatest score is 25.

ED is classified into five severity levels: 22–25 with no ED,17–21 was mild, 12–16 was mild to moderate, 8–11 was moderate, and 1–7 was severe ED. Patients with a score of 21 or below may show indications of ED [8]. It is a validated Arabic version of the IIEF-5 [9].

### EHS Questionnaire

The EHS is a single-item Likert scale that can be used to evaluate ED defined as A man’s difficulty to obtain or preserve a hard erection suitable for intercourse. This tool will prompt them to consider the question, “How would you evaluate the stiffness of your erection?” Select one of the following choices [10]:

### Bariatric Quality of Life (BQoL) Questionnaire

Which was an approved tool created to evaluate patients’ QoL before and after BSs. It comprises 30 questions, separated into two halves. The first component, which consists of 16 items, evaluates non-QoL aspects such as obesity-related co-morbidities, gastrointestinal issues prevalent post-BS, and medication use. The second component, which includes 14 items, measures QoL characteristics by a five-point Likert scale. The BQoL Index score is determined by the summation of all the item scores from both sections, and it runs from 14 to 78, with a higher number signifying better QoL [11].

### Post Bariatric Body Image Satisfaction Questionnaire

Focuses primarily on cases’ satisfaction with their look after weight loss and measures it on a five-point Likert scale (highly satisfied to extremely unsatisfied) [12].

### Hormone Profile Assessment

All studied patients will be subjected to withdrawal of venous blood samples between (8:00–11:00 am) for measurements of Testosterone (Total), Testosterone (Free), and *Estradiol E2* using an electro chemiluminescence immunoassay analyzer [13].

The normal reference values are as follows; Testosterone (Total) = 2.5–8.4 ng/ml, Testosterone (Free) = 46.0–224.0 pg/ml, and E2 less than 42.6 pg/ml).

#### Surgical Procedure

All procedures were done under general anesthesia. After the creation of the pneumoperitoneum LSG was done.

### Post-operative Diet Regimen

A few hours after surgery, patients were urged to move about early. We began administering IV proton pump inhibitors (PPIs) on the day of operation and lasted for 6–8 weeks after the cases began oral eating. Patients began drinking oral fluids on the day of surgery and gradually transitioned to solid diets over the next 6 to 8 weeks.

All cases were recommended to consume oral fluids for the first 15 days, followed by a soft diet for the first month after surgery, and then gradually transition to ordinary meals with an avoidance of fatty foods and high sugar.

All patients were advised to have multivitamins (vitamin B, vitamin D, ca., centrum, whey protein) started in the second week for one year after sleeve and life after gastric bypass.

### Possible Risk

Commonly encountered complications of bariatric surgery (e.g., bleeding, leakage, etc.).

### Primary Outcomes (Most Important Measurable Outcomes)

IIEF-5 Score, EHS, and hormone profile [Testosterone (Total and Free), and Estradiol E2.

### Secondary Outcome Parameters (other Outcomes To Be assessed)

Body mass index (BMI), Bariatric QoL Index, and Post Bariatric Body Image Satisfaction.

### Statistical Analysis

Data were coded and put into SPSS version 28 (IBM Corp., Armonk, NY, USA). Quantitative data was presented as mean, standard deviation, median, minimum, and maximum, while categorical data was described utilizing frequency (count) and relative frequency (%). Comparisons between basal and 6 months values within each patient were performed. For regularly distributed quantitative data, paired t-tests were employed; for non-normally distributed quantitative variables, the non-parametric Wilcoxon signed rank test was employed [14]. For comparing categorical data (baseline and after 6 months), the marginal homogeneity test was used [15]. Correlations between quantitative variables were done by the Spearman correlation coefficient [16]. P-values < 0.05 were statistically significant.

## Results

In our study, the mean age of cases was 44.29 ± 6.40 years. Table (1) showed a statistically significant improvement in BMI after 6 months of operation (P-value < 0.001).

**Table 1:** Comparison of BMI, Weight, BMI, IEF-5, EHS, hormones and bariatric quality of life index score at baseline and after 6 months

	Baseline					After 6 months of operation					*P* value
	Mean	SD	Median	Min	Max	Mean	SD	Median	Min	Max	
Weight	143.50	14.88	141.00	125.00	180.00	94.06	12.37	90.50	80.00	133.00	< 0.001
Height	172.63	5.05	173.00	163.00	180.00	172.63	5.05	173.00	163.0	180.00	---
BMI	48.31	6.25	46.28	40.12	62.86	31.64	4.61	30.65	26.12	46.02	< 0.001
Testosterone total (ng/ml)	2.64	1.15	2.41	1.29	5.30	5.79	2.53	5.05	2.58	10.50	< 0.001
Testosterone free (pg/ml)	43.23	25.04	38.90	16.00	114.00	65.40	36.04	60.25	13.37	170.00	< 0.004
Estradiol (pg/ml)	44.14	16.55	41.45	28.80	99.00	29.94	15.15	25.10	16.80	73.30	< 0.002
Iief- 5 score	14.37	1.83	14	12.00	19.00	21.31	2.12	21.00	19.00	25.00	0.001
EHS score	2.38	0.61	2.00	1.00	3.00	3.50	0.51	3.50	3.00	4.00	0.001
Bariatric quality of life score	41.28	3.11	40.50	36.5	49.50	66.34	3.47	66.00	60.50	73.00	< 0.001

There was a statistically significant improvement in patient IIEF-5 score grade after 6 months of operation (*P* < 0.001). Table (2).

There was a statistically significant improvement in IEF-5 and the Erection Hardness Score after 6 months of operation (*P* < 0.001). There was a statistically significant increase in levels of testosterone (Total) (*P* < 0.001), testosterone (Free) (*P* = 0.005), and decreasing levels of estradiol (E2) (*P* = 0.001) after 6 months of operation. Table (3).

**Table 2:** Effect of LSG on the International Index of Erectile Function (IIEF-5) score grade

		Baseline	After 6 months ofoperation	P value
		Count (%)	Count (%)	
IIEF-5 score at baseline and after 6 months of operation	No ED	0(0.00%)	12(37.50%)	<0.001
	Mild ED	4(12.50%)	20(62.50%)	
	Mild to moderate ED	28(87.50%)	0(0.00%)

**Table 3:** Correlation between IIEF-5, EHS and sex hormones at 6 months of operation

		IIEF-5 SCORE at 6 months	EHS SCORE at 6 months
**Testosterone total at 6 months**	**Correlation coefficient**	0.260	0.298
	**P value**	0.151	0.097
**Testosterone free for 6 months**	**Correlation coefficient**	0.324	0.217
	**P value**	0.070	0.233
**Estradiol at 6 months**	**Correlation coefficient**	0.140	0.163
	**P value**	0.443	0.374

N=42

There was a statistically significant positive association between IIEF-5 and testosterone (Free) (P-value 0.015). In addition, there was a statistically significant positive association between EHS and testosterone (Total and Free) (P-values of 0.015, 0.040, and 0.031). On the other hand, there was no correlation between estradiol and IIEF-5 or EHS at 6 months of operation.

There was a statistically significant adverse association between weight (Baseline) and IIEF-5 after 6 months of operation (P-value 0.040). However, there was no association between weight (baseline) and sex hormones or EHS after 6 months of operation. Table (4).

**Table 4:** Correlation between weight (Baseline) with sex Hormones, IIEF-5 and EHS after 6 months

		Testosterone total after 6 months	Testosterone free after 6 months	Estradiol after 6 months	TT/E2 ratio after 6 months	IIEF-5 score after 6 months	EHS score after 6 months
**Weight** **baseline**	**Correlation coefficient**	0.339	0.385	0.242	−0.131-	−0.122-	0.014
	**P value**	0.057	0.029	0.182	0.474	0.505	0.941

N=42

There was a statistically significant improvement in the BQoL Index after 6 months of operation (P-value < 0.001). There was a statistically significant improvement in Bariatric Body Image Satisfaction (general, upper arms, abdomen, breast, thighs, and buttocks) after 6 months of operation (P-value < 0.001). Table (5).

**Table 5:** Descriptive of bariatric body image satisfaction score values at baseline and six months postoperative

	Baseline					After 6 months					*P* value
	Mean	SD	Median	Min	Max	Mean	SD	Median	Min	Max	
**General satisfaction score**	1.87	0.71	2.00	1.00	3.00	4.75	0.44	5.00	4.00	5.00	< 0.001
**Upper arms satisfaction score**	1.81	0.64	2.00	1.00	3.00	4.31	0.59	4.00	3.00	5.00	< 0.001
**Abdomen satisfaction score**	1.00	0.00	1.00	1.00	1.00	3.56	0.72	4.00	2.00	5.00	< 0.001
**Breast satisfaction score**	1.38	0.49	1.00	1.00	2.00	4.25	0.98	5.00	2.00	5.00	< 0.001
**Thighs satisfaction score**	1.37	0.61	1.00	1.00	3.00	4.44	0.62	4.50	3.00	5.00	< 0.001
**Buttock satisfaction score**	1.06	0.25	1.00	1.00	2.00	4.13	0.87	4.00	3.00	5.00	< 0.001

## Discussion

Obesity is caused by calorie intake exceeding over time, although its factors are complex and diverse. The human body has fine-tuned processes for maintaining body weight equilibrium. However, these processes frequently fail for a variety of reasons, including genetic, metabolic, hormonal, psychological, physical, and social abnormalities.

Obesity causes the onset of psychosocial disorders and a vast set of comorbidities, which are connected with a lower QoL and have an adverse influence on the healthcare system [17].

Obesity has multiple effects on the capacity to get and sustain an erection [18]. Obesity and ED in males appear to be linked in studies. Obesity is a significant risk factor for the progress of ED, which is a serious clinical issue [19].

Multiple investigations have found that abnormal deposition of adipose tissue has an adverse impact on reproductive functioning in both men and women [20]. Approximately 40% of obese men acquire Male Obesity Secondary Hypogonadism (MOSH). Obesity in males reduces testosterone and sex hormone-binding globulin (SHBG) while increasing estradiol [21]. Obesity impacts not just the level of sex hormones, but additionally the entire sexual health [19].

This prospective observational study included 32 morbidly obese male patients who underwent LSG for weight reduction.

Concerning the effect of BS on erectile function, Xu J. et al. [6] revealed that BS led to a remarkable elevation in the IIEF-5 score after BS. Lee et al. [22] highlighted the findings on the impact of BS on sexual function and male sex hormones. The review findings showed an increase in erectile function, and these results are the same as our study.

Efthymiou et al. [23] published a study that included 80 severely obese cases, 30 of whom were men. The IIEF and QoL were assessed before and following BS. Erectile function improved from before surgery to 1 year postoperative, with the greatest improvement observed 1 to 6 months following surgery. In addition, bariatric surgery significantly improved patients’ QOL. Recently, Sarhan et al. [24] also conducted a study that included 48 patients who completed the study. Results showed that the patients had a highly significant increase in the IIEF scores (*p* < 0.001) at T2. Moreover, weight was a significant predictor of the IIEF scores, and these results are the same as our study.

On the other hand, Sarwer et al. [25] obtained findings that were inconsistent with the current study which demonstrated a non-significant difference in the enhancement of sexual functioning, particularly erectile function in males with obesity after BS. Nevertheless, Legro et al. [26] noticed no substantial change in erectile function and only detected an improvement 12 months following surgery.

Concerning the effect of BS on sex hormones, Lee et al. [22] assessed the effects of BS on male sex hormones. The evaluation results revealed a rise in the levels of sex hormones TT, LH, FSH, and SHBG. In contrast, estradiol and prolactin levels were significantly decreased after BS. The results of this study showed similar results to ours regarding increasing testosterone levels and decreasing estradiol levels. Sarhan et al. [24] also reported an increase in serum testosterone levels (*p* < 0.001) postoperatively. These results are the same as our study.

Aarts et al. [27] also reported a rise in serum testosterone (total) and testosterone (free); however, serum E2 and SHBG levels did not change significantly, and this was not in line with our study that showed a decrease in estradiol.

Regarding the impact of BS on QoL, Lindekilde et al. [28] the influence of BS on QoL and found that it had a considerable helpful effect on QoL in general. Efthymiou et al. [23] also reported that bariatric surgery significantly improved patients’ QOL, and these results are similar to our study.

Regarding the effect of BS on post-bariatric body image satisfaction, Alamri et al. [29] performed an investigation to evaluate post-BS body image satisfaction after weight loss among post-bariatric patients. The research found that the majority of cases who received BS were satisfied with their body image, except for their abdomen. Compared to our study, the findings of this study agreed with ours, which showed improvement in all regions.

The study’s limitations include a short follow-up period, a small study sample, and a lack of assessment of additional metabolic variables and anthropometric parameters that could have influenced the study outcome.

## Conclusion

Morbidly obese males who underwent BS showed a considerable improvement in erectile performance. This enhancement was clinically demonstrated by a higher IIEF-5 score. Bariatric surgeries have a direct effect on serum sex hormones by decreasing E2 and increasing Testosterone (Total and Free) levels. BS is effective in improving QoL and Post Bariatric Body Image Satisfaction.

### Limitation of the Study

Limitations include a short follow-up period and a small sample size as all men in our study from Eastern society. Where there was embarrassment in talking about their sexual life and answering our study questionnaires.

## Supplementary Information

Below is the link to the electronic supplementary material.

Supplementary Material 1

## Data Availability

Data is available upon reasonable request from corresponding author.
