# Supplementary material for: The Influence of Laparscopic Sleeve Gastrectomy on Male Erectile Function among Morbid Obese Patients: an Observational Study
Source: Obes Surg. 2025 Dec 4;36(1):98–104. doi: 10.1007/s11695-025-08376-5 (PMC12852243; doi:10.1007/s11695-025-08376-5)
Supplement: Supplementary file 1 — Supplementary Material 1 [file 11695_2025_8376_MOESM1_ESM.pptx]

## Slide 1
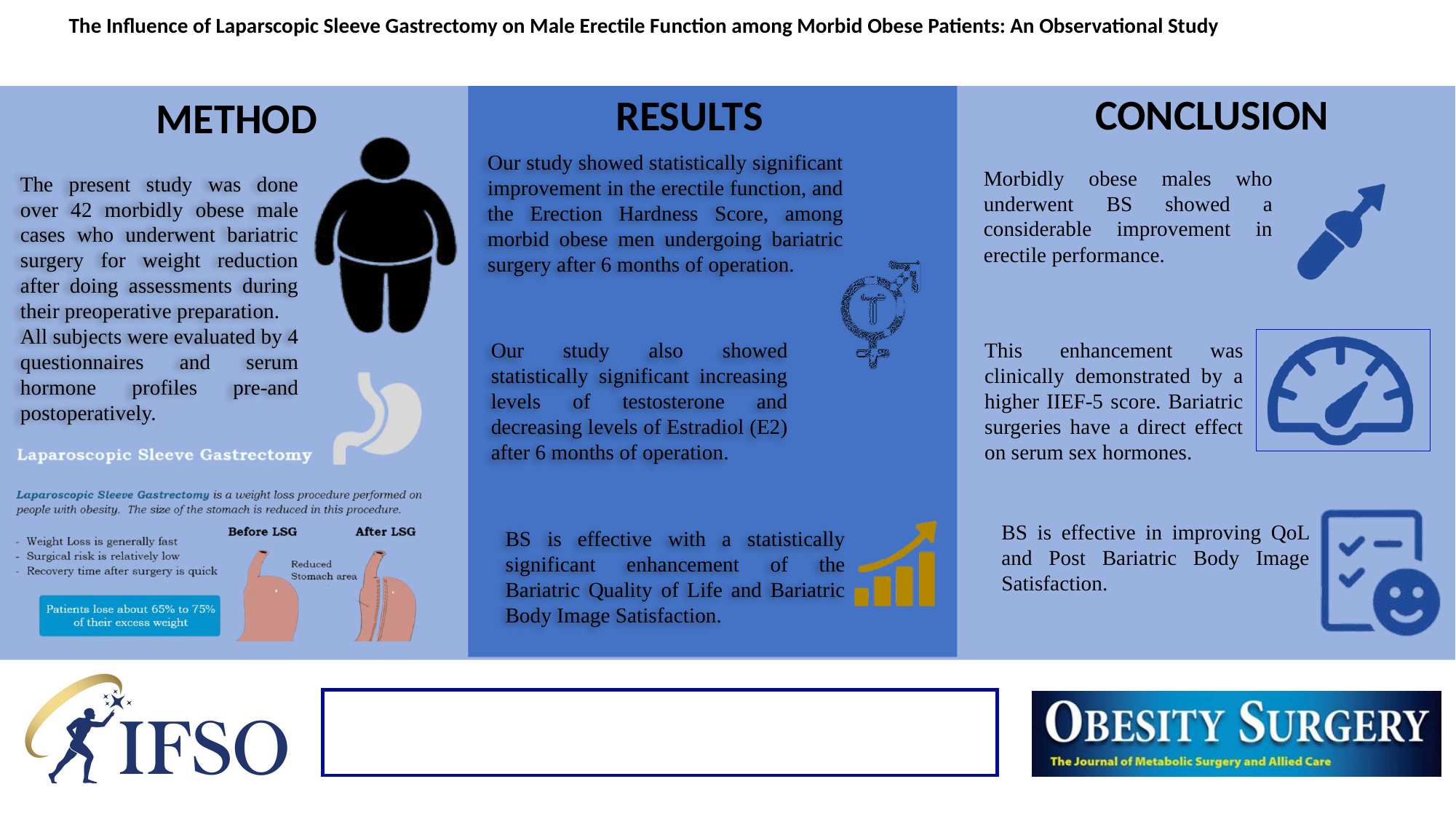

The Influence of Laparscopic Sleeve Gastrectomy on Male Erectile Function among Morbid Obese Patients: An Observational Study
CONCLUSION
RESULTS
Conclusion
 METHOD
Our study showed statistically significant improvement in the erectile function, and the Erection Hardness Score, among morbid obese men undergoing bariatric surgery after 6 months of operation.
Morbidly obese males who underwent BS showed a considerable improvement in erectile performance.
The present study was done over 42 morbidly obese male cases who underwent bariatric surgery for weight reduction after doing assessments during their preoperative preparation.
All subjects were evaluated by 4 questionnaires and serum hormone profiles pre-and postoperatively.
Our study also showed statistically significant increasing levels of testosterone and decreasing levels of Estradiol (E2) after 6 months of operation.
This enhancement was clinically demonstrated by a higher IIEF-5 score. Bariatric surgeries have a direct effect on serum sex hormones.
BS is effective in improving QoL and Post Bariatric Body Image Satisfaction.
BS is effective with a statistically significant enhancement of the Bariatric Quality of Life and Bariatric Body Image Satisfaction.
